# Nutritional Composition and Antioxidant Properties of *Cucumis dipsaceus* Ehrenb. ex Spach Leaf

**DOI:** 10.1155/2013/890451

**Published:** 2013-10-27

**Authors:** Rahul Chandran, V. Nivedhini, Thangaraj Parimelazhagan

**Affiliations:** Bioprospecting Laboratory, Department of Botany, Bharathiar University, Coimbatore, Tamil Nadu 641046, India

## Abstract

The leaf of *C. dipsaceus* was evaluated for its nutritional and antioxidant properties. From the present investigation, significant amount of almost all essential amino acids and important minerals were quantified. Low levels of trypsin inhibitory units, phenolics, and tannins content were found as antinutritional content. Further, hot water extract of *C. dipsaceus* showed good activity especially in ABTS^+^, metal chelating, nitric oxide, and DPPH assays. Hence, the results conclude that *C. dipsaceus* could be a valuable nutraceutical supplement to the human diet.

## 1. Introduction

The consumption of locally grown, wild, or semiwild edible plants has been important for most human cultures [1]. They often contain higher amount of nutrients and bioactive compounds than many cultivated species, especially those which have been under cultivation for many generations [2]. There are certain factors that may play an adverse role in nutritional diet. Antinutrients are such natural or synthetic compounds that interfere with the absorption of nutrients. One common example is “Phytate,” which forms insoluble complexes with calcium, zinc, iron, and copper [3]. Proteins can also be antinutrients, such as the trypsin inhibitor and lectins found in legumes [4]. Flavonoids are a form of anti-nutritional factors. These compounds chelate metals such as iron and zinc and reduce the absorption of these nutrients. They also inhibit digestive enzymes and may precipitate proteins [5]. The investigations of such nutritional and anti-nutritional factors enable us to know the nutritional and anti-nutritional values and to avoid consumption of highly toxic plants. It will also provide knowledge on the nutritional implication of feeding on staples of low nutritive quality, which will help to ensure better health condition of people in developing countries [3].

Free radicals are highly unstable and undergo chemical reactions either to grab or donate electrons, thereby causing damage to proteins, cells, and DNA [6]. However, the presence of free radicals within the body can also have significant role in the development and progression of many disease processes like congestive heart failure, hypertension, cerebrovascular accidents, and diabetic complications [7]. Degradation due to oxidative reactions can affect all biomolecules, but mostly lipids, carbohydrates, and proteins [8]. Synthetic antioxidants like butylated hydroxyl anisole (BHA) and butylated hydroxyl toluene (BHT) have been restricted in foods, as they are suspected to be carcinogenic [9, 10]. So, the interest is highly focussed on searching plant based antioxidants because of their therapeutic performance and low toxicity. Antioxidants protect the integrity of cellular structures and macromolecules from damage due to free radicals. Carotenoids and phenolic compounds are dietary antioxidants [11].

*Cucumis dipsaceus* Ehrenb. ex Spach is a species of flowering plant belonging to the family Cucurbitaceae. It has its origin in Ethiopia. It is known by several common names like “teasel gourd, Arabian cucumber, hedgehog, pepino-diablito, concombre porc-epic, and so on.” Usually, the leaves of *Cucumis dipsaceus* are consumed as a leafy vegetable [12]; its fruit juice is topically applied to prevent hair loss [13]. The cooked plant is also consumed in Kenya [14]. Hence, this is the first attempt to evaluate wild leafy vegetable *C. dipsaceus *for nutritional and antioxidant properties.

## 2. Materials and Methods

### 2.1. Collection of Plant Materials

The leaves were collected during the month of November 2011. The collected plant material was identified, and their authenticity was confirmed by comparing the voucher specimen at the Herbarium of Botanical Survey of India, Southern Circle Coimbatore, Tamil Nadu. Freshly collected plant material was cleaned to remove adhering dust and then dried under shade. The dried sample was powdered and used for further studies.

### 2.2. Chemicals

Potassium ferricyanide, ferric chloride, 2,2-diphenyl-1-picryl-hydrazyl (DPPH), potassium persulfate, 2,2'azinobis(3-ethylbenzothiozoline-6-sulfonic acid) diammonium salt (ABTS), 6-hydroxy-2,5,7,8-tetramethylchroman-2-carboxylic acid (Trolox), linoleic acid, ferrous chloride, ammonium thiocyanate, 2,4,6-tripyridyl-*s*-triazine (TPTZ), polyvinyl polypyrrolidone (PVPP), hydrogen peroxide, ferrous ammonium sulphate, ethylenediamine tetraacetic acid (EDTA) disodium salt, 2,2′-bipyridyl and hydroxylamine hydrochloride, metaphosphoric acid, 2, 6-dichoroindophenols, trypsin, and BAPNA were obtained from Himedia (Mumbai, Maharashtra, India), Merck (Hyderabad, Andhra Pradesh, India), and Sigma (Thane, Maharashtra, India). All other reagents used were of analytical grade.

### 2.3. Successive Solvent Extraction

The air dried, powdered plant material was extracted in soxhlet extractor successively with petroleum ether and methanol. Finally, the material was macerated using hot water with occasional stirring for 24 hr, and the water extract was filtered. The methanol extract alone was subjected to fractional extraction using chloroform, ethyl acetate, and methanol. Each time before extracting with the next solvent, the material was dried in hot air oven below 40°C. The different solvent extracts were concentrated by rotary vacuum evaporator and then air dried. The dried extract obtained with each solvent was weighed. The percentage yield was expressed in terms of air dried weight of plant material. The chloroform, ethyl acetate, methanol, and hot water extracts thus obtained were used directly for the estimation of phytochemical screening, total phenolics, and also for the assessment of antioxidant potential through various biochemical assays. The extracts were freeze-dried and stored in desiccators until further analysis.

### 2.4. Qualitative Phytochemical Analysis

Leaves were analyzed for the presence of major phytochemicals such as carbohydrates, proteins, amino acids, alkaloids, saponins, phenolic compounds, tannins, flavonoids, glycosides, flavanol glycosides, cardiac glycosides, phytosterols, fixed oils and fats, and gums and mucilages according to standard methods such as Hager's test, the Frothing test, Borntrager's test, the Keller-Kiliani test, Libermann and Burchard's test, and the Saponification test [15].

### 2.5. Nutritional Analysis

#### 2.5.1. Proximate Composition

The moisture content of the leaf was estimated by taking plant samples, and the weight was taken before and after incubation in a hot-air-oven at 50°C for 24 h, followed by cooling in a desiccator. The recommended methods of Association of Official Analytical Chemists [16] were used for the determination of ash. Ash content was determined by incineration of 2 g of sample in a muffle furnace kept at 600°C for 6 h.

#### 2.5.2. Determination of Total Proteins

The protein was estimated as described by Lowry et al. [17] using Bovine Serum Albumin as a standard. 100 mg of sample powder was ground with 10 mL of phosphate buffer in mortar and pestle. Then, the filtrate was centrifuged at 5000 rpm for 5 minutes. The supernatant was used for further analysis. Reagent A: 2% sodium carbonate in 0.1 N sodium hydroxide, reagent B: 1% sodium potassium tartrate with 0.5 gm of CuSO_4_, reagent C: 200 mL of reagent A was added with 4 mL of the reagent B which was mixed prior to use, and reagent D: Folin-Ciocalteu's reagent was used. Bovine serum albumin was used as a standard (0.01 g of BSA in 10 mL of distilled water). A 0.5 to 1 mL of diluted supernatant (10^−1^) was added in to the test tubes and it was made up to 100 mL with distilled water. Then, 5 mL of reagent C was added. To this, 0.5 mL of reagent D was added and was allowed to incubated in dark for 30 minutes, and the absorbance was determined at 660 nm using spectrophotometer.

#### 2.5.3. Determination of Total Carbohydrates

The carbohydrate was estimated as described by Sadasivam and Manikam [18] using Glucose as a standard. 100 mg of sample powder was ground with 10 mL of 80% acetone in mortar and pestle. Then, the filtrate was centrifuged at 5000 rpm for 5 minutes. The supernatant was used for further analysis. 400 mg of anthrone reagent was dissolved in 190 mL of ice-cold concentrated sulphuric acid with 10 mL of distilled water. Glucose was used as a standard. 10 mg of glucose was dissolved in 100 mL of distilled water. A 0.5 to 1 mL of diluted supernatant (10^−1^) was taken in the test tubes. It was made up to 1 mL with distilled water. A 4 mL of anthrone reagent was added. The tubes were treated over a boiling water bath for 10 minutes and then cooled down to room temperature. The absorbance of a blue green solution was measured at 630 nm using spectrophotometer and compared with a standard curve preparation with known amounts of glucose. The amount of total carbohydrate present in each sample was calculated and the results were tabulated.

#### 2.5.4. Estimation of Amino Acids

Amino acids in leaves were determined according to the procedure of Ishida et al. [19]. Extracted samples were filtered through a 0.45 *μ*m membrane, filter, and 20 *μ*L of the filtrate was injected in to a HPLC (model LC 10 AS, Shimadzu, Mount holly, New Jersey) equipped with a cation exchange column packed with a strongly acidic cation exchange resin, that is, styrene divinylbenzene copolymer with sulphonic group. The amino acid analysis was with the nonswitching flow method and fluorescence detection after postcolumn derivatization with o-phthalaldehyde. Amino acid standards were used to calculate amino acid concentrations in samples.

#### 2.5.5. Mineral Quantification

For the determination of mineral contents in the sample, digestion mixture was prepared following standard method. For digestion, 0.5 g of dried sample was mixed with 5 mL digestion mixture and kept in digestion unit at 300°C. The process was allowed to continue till the mixture turns colourless. Desired volume of distilled water is added to the digested and cooled samples. Solution was filtered and mixed well till all sediments got dissolved. Subsequently, minerals were determined as follows: nitrogen (N) through micro-Kjeldahl method; phosphorus (P) by treating the digested samples with ammonium molybdate and freshly prepared ascorbic acid and analyzed by spectrophotometer (Hitachi U-2001 Japan); potassium (K), sodium (Na), and calcium (Ca) were determined by Flame Photometer by the method of Allen [20]. The microelements (Fe, CO, Cu, Mg, Mn, and Zn) were determined through atomic absorption spectrophotometer.

#### 2.5.6. Analysis of Antinutritional Factors

Trypsin inhibition ability was evaluated in powdered leaf samples on a synthetic substrate BAPNA. The degree of inhibition is expressed in TIU/mg protein by the method of Sadasivam and Manikam [18]. Total phenolics [21] and Tannin content [22] were also determined.

### 2.6. *In Vitro* Antioxidant Studies

#### 2.6.1. Quantification of Total Phenolics, Tannins, and Flavonoids

The total phenol content was determined according to the method described by Siddhuraju and Becker [21]. 200 *μ*L triplicate for both fruit and leaf extracts (2 mg/2 mL) was taken in the test tubes and made up to the volume of 1 mL with distilled water. Then, 0.5 mL of Folin-Ciocalteu reagent (1 : 1 with water) and 2.5 mL of sodium carbonate solution (20%) were added sequentially in each tube. Soon after vortexing the reaction mixture, the test tubes were placed in dark for 40 min and the absorbance was recorded at 725 nm against blank. Reaction mixture without plant extract was taken as blank. The analysis was performed in triplicate and the results were expressed as gallic acid equivalents.

Using the same extract, the tannins were estimated after treatment with polyvinyl polypyrrolidone (PVPP) Siddhuraju and Manian [22]. 100 mg of PVPP was weighed into a 100 × 12 mm test tube and to this 500 *μ*L distilled water, and then 500 *μ*L of the sample extracts were added. The content was vortexed and kept in the test tube at 4°C for 15 min. Then, the sample was centrifuged at 4000 rpm for 10 min at room temperature and the supernatant was collected. This supernatant has only simple phenolics other than the tannins (the tannins would have been precipitated along with the PVPP). The phenolic content of the supernatant was measured and expressed as the content of nontannin phenolics. From the above results, the tannin content of the sample was calculated as follows:

(1) Tannin(%) =Total  phenolics(%)−Non  tannin  phenolics(%).

The flavonoid contents of all the extracts were quantified as they act as a major antioxidant in plants reducing oxidative stress, estimated as described by Zhishen et al. [23]. Initially, 700 *μ*L of all the plant extracts was taken in different test tubes. To each extract 2 mL of distilled water was added. Then, 150 *μ*L of NaNO_2_ was added to all the test tubes followed by incubation at room temperature for 6 minutes. After incubation, 150 *μ*L of AlCl_3_ (10%) was added to all the test tubes. The test tubes were incubated for 6 minutes at room temperature. Then, 2 mL of 4% NaOH was added to all the test tubes which were made up to 5 mL using distilled water. The contents in all the test tubes were vortexed well and they were allowed to stand for 15 minutes at room temperature. The pink colour developed due to the presence of flavonoids was read spectrophotometrically at 510 nm. The amount of flavonoid was calculated in rutin equivalents.

#### 2.6.2. Total Antioxidant Activity Assay by Radical Cation 2, 2′-Azinobis(3-ethylbenzothiozoline-6-sulfonic acid) (ABTS^•+^)

ABTS radical cation decolorization assay was done to determine the total antioxidant activity of the samples according to the method of Re et al. [24] described by Siddhuraju and Manian [22]. ABTS^•+^ was produced by reacting 7 mM ABTS^•+^ aqueous solution with 2.4 mM potassium persulphate in the dark for 12–16 hr at room temperature. The reagent solution was diluted in ethanol (about 1 : 89 v/v) and equilibrated at 30°C to give an absorbance at 734 nm of 0.7 ± 0.02. After the addition of 1 mL of diluted ABTS^•+^ solution to different concentrations of sample or trolox standards (final concentration 0–15 *μ*M) in ethanol, absorbance was measured at 30°C exactly 30 min after initial mixing. Triplicate determinations were made at each dilution of the standard, and the percentage inhibition was calculated of the blank absorbance at 734 nm, and it was plotted as a function of trolox concentration. The unit of total antioxidant activity (TAA) is defined as the concentration of trolox having equivalent antioxidant activity expressed as *μ*Mol/g extract.

#### 2.6.3. Radical Scavenging Activity Using DPPH^•^ Method

The antioxidant activity of the extracts was determined in terms of hydrogen donating or radical scavenging ability, using the stable radical 2,2-diphenyl-2-picrylhydrazyl(DPPH^•^), according to the method of Blois [25]. A methanol solution of the sample extracts at various concentrations was added to 5 mL of 0.1 mM methanolic solution of DPPH^•^ and allowed to stand for 20 min at 27°C. The absorbance of the sample was measured at 517 nm. Methanol was served as blank, and solution without extract served as control. The mixture of methanol, DPPH, and standard (BHT, BHA, quercetin, and *α*-tocopherol) served as positive control. More significantly, the IC_50_ of the extracts were also calculated.

#### 2.6.4. Ferric Reducing Antioxidant Power (FRAP) Assay

The antioxidant capacities of phenolic extracts of samples were estimated according to the procedure described by Pulido et al. [26]. Freshly prepared FRAP reagent (2.5 mL of 20 mmol/L TPTZ (2,4,6-tripyridyl-*s*-triazine) solution in 40 mmol/l HCl plus 2.5 mL of 20 mmol/L FeCl_3_·6H_2_O and 25 mL of 0.3 mol/L acetate buffer (pH 3.6)) (900 *μ*l) incubated at 37°C was mixed with test sample or methanol (for the reagent blank). The test samples and reagent blank were incubated at 37°C for 30 min in a water bath as described by Siddhuraju and Becker [21]. At the end of incubation, the absorbance readings were taken immediately at 593 nm. Results were calculated in ascorbic acid equivalents.

#### 2.6.5. Metal Chelating Activity

The chelating of ferrous ions by leaf and barkextracts was estimated by the method of Dinis et al. [27]. Briefly, 50 *μ*l of 2 mM FeCl_2_ was added to the extracts. The reaction was initiated by the addition of 0.2 mL of 5 mM ferrozine solution. The mixture was vigorously shaken and left to stand at room temperature for 10 min. The absorbance of the solution was thereafter measured at 562 nm. BHT was taken as standard. All the reagents without addition of sample extract were used as negative control. Metal chelating activity was determined in EDTA equivalence.

#### 2.6.6. Nitric Oxide Radical Scavenging Activity

The procedure is based on the method, where sodium nitroprusside in aqueous solution at physiological pH spontaneously generates nitric oxide, which interacts with the oxygen to produce nitrite ions that can be estimated using Griess reagent. Scavengers of nitric oxide compete with oxygen leading to reduced production of nitrite ions. The nitric oxide scavenging activity of different solvent extracts of *C. dipsaceus *on nitric oxide radical was measured according to the method of Sreejayan and Rao [28]. Sodium nitroprusside (10 mM) in phosphate buffered saline, was mixed with different concentrations (50–250 *μ*g) of *C. dipsaceus *extracts and incubated at room temperature for 150 min. Griess reagent (0.5 mL) containing 1% sulphanilamide, 2% H_3_PO_4_, and 0.1% N-(1-naphthyl) ethylene diamine dihydrochloride was added to the mixture after incubation time. The absorbance of the chromophore formed was read at 546 nm. BHT and rutin and the same mixture of the reaction without *C. dipsaceus *extracts were employed as positive and negative control. Radical scavenging activity was expressed as the inhibition percentage of free radical by the sample and was calculated using the following formula:

(2) %radical  scavenging  activity =(Control  OD−Sample  ODControl  OD)×100.

#### 2.6.7. Assay of Superoxide Radical (*O*_2_^•^^−^) Scavenging Activity

The ability to inhibit formazan formation by scavenging superoxide radicals by the extracts was studied by the method of Beauchamp and Fridovich [29]. Each 3 mL reaction mixture (50 mM sodium phosphate buffer (pH 7.6), 20 *μ*g riboflavin and 12 mM EDTA, and 0.1 mg NBT) with extracts in each test tubes was illuminated for 90 s. Illuminated reaction mixture served as negative control, while the mixture without extract in dark was taken as blank. Immediately after illumination, the absorbance was measured at 590 nm. The activity was compared to BHT and BHA. The percentage inhibition of superoxide anion generation was calculated using the following formula:

(3) $$\begin{array}{c} \%\text{inhibition}=\left(\;\frac{\text{Control}\;\text{OD}-\text{Sample}\;\text{OD}}{\text{Control}\;\text{OD}}\right)\times 100. \end{array}$$

#### 2.6.8. Phosphomolybdenum Assay

The antioxidant activity of samples was evaluated by the phosphomolybdenum method [30]. Sample solution was combined with 1 mL of reagent solution (0.6 M sulphuric acid, 28 mM sodium phosphate, and 4 mM ammonium molybdate). The reaction mixture was incubated in a water bath at 95°C for 90 min. After cooling to room temperature, the absorbance of the mixture was measured at 765 nm against a blank. The results were reported in ascorbic acid equivalents per gram extract (AEAC).

### 2.7. Statistical Analysis

The results were statistically analyzed and expressed as mean (*n* = 3)  ± standard deviation. Values are analyzed by Duncan's multiple test range (SPSS, ANNOVA statistical software, TULSA, USA).

## 3. Result and Discussion

### 3.1. Qualitative Phytochemical Analysis

Preliminary phytochemical screening was done for the qualitative phytochemical profiling in *C. dipsaceus. *The screening test for carbohydrates, proteins, amino acids, alkaloids, saponins, phenolics, tannins, glycosides, fats and oils, flavonols glycosides, and phytosterols was done and the results were shown in Table 1. The results revealed that the plant has potential phytochemicals with important biological activities. These phytochemicals also indicate the richness medicinal value in leaf.

### 3.2. Moisture and Ash Content

The moisture content of the leaf was determined by calculating its initial and final weight. After 2 days of hot air oven treatment under 60°C, moisture content of leaf was found to be having 83%. The ash content of leaf is 10%.

### 3.3. Nutritional Evaluation

In the quantification of amino acids and minerals, it was found that almost all essential amino acids are present in an appreciable amount in the leaf sample. The amount of glutamic acid is higher (10.72%), whereas the amount of glutamine and tryptophan could not be detected. It was reported that for a healthy human diet, a normal man should take 15 mg of threonine, 4 mg of cysteine, 10 mg of methionine, 26 mg of valine, 20 mg of isoleucine, 39 mg of leucine, 15 mg of tyrosine, 10 mg of histidine, 25 mg of phenylalanine, and 30 mg of lysine per kg/day of body weight [31]. The results are shown in Table 2(a). Leucine, isoleucine, alanine, and valine enhance muscular energy production, stimulate metabolic signals, and are precursors of several other amino acids. Amino acids are also reported to have the property of quenching the deleterious 2,2-diphenyle-1-picrylhydrazyl (DPPH) radical also [32, 33]. The chemical score and amino acid index are widely used for screening potential protein foods. It was reported that the amino acid deficiency can be met by consuming large amounts of legumes, by employing the complimentarily that exists between high sulphur amino acid substitutes [34]. Since the plant *C. dipsaceus* is observed to be having those amino acids in sufficient quantities, it can be justified as a promising amino acid source. Moreover, the amino acid content of the plant could also reflect the protein richness and its role in human diet.

Based on the evidences for the edible property [12], leaves of *C. dipsaceus* were quantified for the presence of important macro- and micronutrients, and the results are presented in Table 2(b). In the analysis of macro- and micronutrients, the leaf sample is found to have N, K, Na, Ca, P, and Fe in a well-appreciable amount. In leaf, the calcium content (27000 ppm) is estimated to be higher than all the other macro- and micronutrients. Magnesium (15300 ppm) is required by many enzymes, in particular the sugar and protein kinase families of enzymes that catalyze ATP-dependent phosphorylation reactions [35]. Manganese is an essential trace metal found in all tissues and is required for normal amino acid, lipid, protein, and carbohydrate metabolism [36]. Calcium is important in bone, teeth, and muscle metabolism [37]. Since the calcium and iron form the important part of our daily diet as they play a major role in strengthening of bones and haemoglobin formation, leaves of the plant *C. dipsaceus* can be recommended as a dietary supplement. It has been well studied that lack of some essential minerals in food may lead to malnutrition and diseases causing serious health problems. Hence, the plant like *C. dipsaceus* could be a good source of minerals that can supplement dietary minerals in food. Beside Cu and Fe as main minerals, it can also serve as a accessory source of other minerals.

Apart from the minerals and amino acids, the leaf of *C. dipsaceus* shows commendable presence of starch and proteins which are essential in daily human diet. The leaf is found to have 0.87 mg/g of starch and 108.2 mg/g of proteins. It was reported that the macronutrients are essential for proper functioning of cell and cellular organs and they act mainly as electrolytes [38]. It was also reported that the trace elements are mainly involved in catalytic activity [39]. From the nutritional studies, it can be established that *C. dipsaceus* can form a promising source of both minerals and amino acids. *C. dipsaceus* could be recommended for use as one among the major leafy vegetables available in the market.

### 3.4. Analysis of Antinutritional Factors

Leaf samples showed 15.61% of phenolics and 10.29% of tannins. 2.36 TIU/mg protein was determined from the leaf sample. The negative nutritional effects of tannins are diverse and incompletely understood, but the major effect is to cause growth depression by decreasing the digestibility of protein and carbohydrate. This is most likely the consequence of the interaction of tannins with either protein or starch to form enzyme-resistant substances [40]. Trypsin inhibitors ingested in significant amounts disrupt the digestive process and may lead to undesirable physiological reactions. Trypsin inhibitor is thermolabile and its inhibitory activity can be reduced considerably by thermal treatment [40]. The negligible presence of antinutritional factors should not pose a problem to human health if leaves and flowers are properly processed. Levels of phenolics and tannin can be reduced by simple processing methods including soaking, roasting, and autoclaving.

### 3.5. *In Vitro* Antioxidant Assays

#### 3.5.1. Quantification of Total Phenolics, Tannins, and Flavonoids

Table 3 shows the total phenolics, tannins, and flavonoid content of *Cucumis dipsaceus *leaf. The chloroform extract of leaf showed maximum phenolic content (5.81 g GAE/100 g extract), and the least amount was detected in ethyl acetate extract (1.66 g GAE/100 g extract). On the other hand, higher tannin content was observed in ethyl acetate extract (13.69 g GAE/100 g extract).

It had been reported that the antioxidant activity of plant materials was well correlated with the content of their phenolic compounds [41]. It was also demonstrated that in *Olea europaea*, there is a significant antioxidant activity, which is higher than vitamins C and E, due to the synergy between flavonoids, substituted phenols, and so forth. These studies strongly support that *C. dipsaceus *undoubtedly can have antioxidant and other medicinal property.

#### 3.5.2. Total Antioxidant Activity Assay by Radical Cation 2,2′-Azinobis(3-ethylbenzothiazoline-6-sulfonic acid) (ABTS^•+^)

In leaf, the maximum activity was observed in methanol extract and the least but noticeable activity of chloroform extract. In ABTS radical scavenging, leaf samples were expressed as trolox equivalent. Hagerman et al. [42] have reported that the high molecular weight phenolics (tannins) have more ability to quench free radicals (ABTS^+^). As the total phenolics and tannins in *C. dipsaceus* have been proved, the plant can be suggested for the use in various nutraceuticals. The results of the total antioxidant activity of different extracts are given in Table 4.

#### 3.5.3. Radical Scavenging Activity Using DPPH^•^ Method

**Figure 1:**
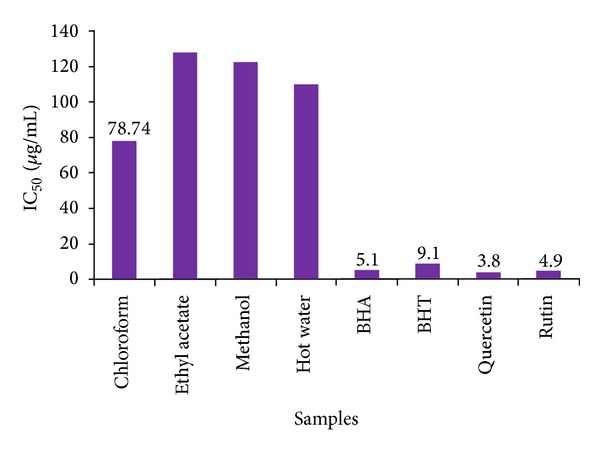
DPPH radical scavenging activity of leaf and fruit extract of *C. dipsaceus*. Values are mean of triplicate determination (*n* = 3)  ±  standard deviation.

The free radical scavenging activity of the leaf extracts of *C. dipsaceus *was estimated by comparing with standards such as BHT, BHA, quercetin, and rutin, and the result is shown in Figure 1. Importantly, IC_50_ value of the extracts was also calculated to determine the amount of extract needed to quench 50% of radicals. A lower value of IC_50_ indicates a higher antioxidant activity. Chloroform extract of leaf (72.74 *μ*g/mL) registered higher DPPH radical scavenging activity, respectively, as compared to other extracts. Even though the radical scavenging activity shown by the extracts was low when compared to synthetic antioxidants like BHT and BHA, coming to the point of safety, it can be prescribed as a safe antioxidant source, as the synthetic antioxidant is reported to pose certain side-effects.

A related species of *C. dipsaceus*, *Cucumis melo*, was reported to possess the highest DPPH radical scavenging activity in its methanolic seed extract which was found to be 75.59% at concentration of 300 *μ*g ML^−1^ [43]. As the inhibition percentage of *C. dipsaceus* leaves extract also showed appreciable activity against DPPH, it can be prescribed as a safe and economical antioxidant source.

#### 3.5.4. Ferric Reducing Antioxidant Power (FRAP) Assay

The results presented in Table 4 show that hot water extract of leaf can be appreciated for its higher (294.1 *μ*M Fe (II)/mg) activity over the other extracts showing activity in the order methanol > chloroform > ethyl acetate. The FRAP assay measures the antioxidant effect of any substances in the reaction medium as reducing ability. The efficiency of antioxidant property depends on the redox potentials of the compound under study [26]. The results are shown in Table 4.

#### 3.5.5. Metal Chelating Activity

The Fe^2+^ chelating activity of extracts is shown in Table 4. The maximum chelation was observed in leaf hot water extract (11.06 g EDTA equi/100 g extract). Ethyl acetate extract (0.44 g EDTA equi/100 g extract) showed the least chelation. Iron is an essential element which is necessary for transport of oxygen molecule through blood. But under certain stress conditions, these iron act as harmful free radical which will catalyze oxidative change in lipid, protein, and other cellular components [44] which are needed to be scavenged using efficient antioxidants. Metal chelating ability was significant as they reduce the concentration of catalyzing transition metal in lipid peroxidation [45]. Quantification of EDTA equivalent metal chelator has given a clear indication that these extracts can effectively chelate metal ions thereby reducing the harm of such metal radicals. From these results, it can be observed that *C. dipsaceus* has high ability of radical scavenging activity.

#### 3.5.6. Nitric Oxide Radical Scavenging Activity

**Figure 2:**
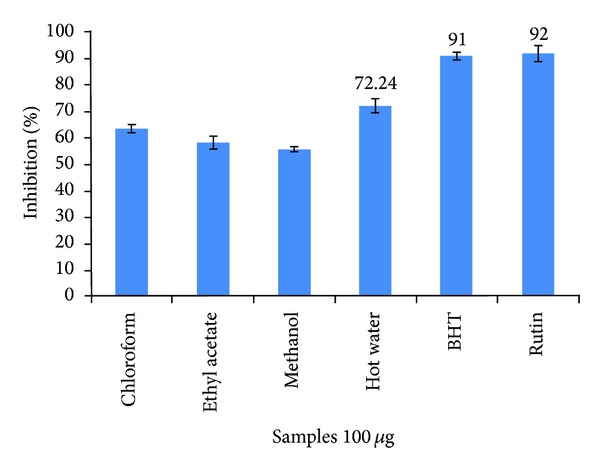
Nitric oxide radical scavenging activity of leaf extract of *C. dipsaceus*. Values are mean of triplicate determination (*n* = 3)  ±  standard deviation.

From Figure 2, it was observed that the scavenging percentage of nitric oxide was higher in leaf hot water extract (72.24%) and lower in methanolic extract (55.70%). It was concluded that all the extracts are likely to have nitric oxide scavenging activity which is shown in Figure 2. The activity was in the order hot water > chloroform > ethyl acetate > methanol. So, it can be interpreted that the plant has the property to counteract the harmful effects of NO and other reactive nitrogen species (RNS). With these lines of evidence, it was anticipated that *C. dipsaceus *has also showed scavenging activity against nitric oxide.

#### 3.5.7. Assay of Superoxide Radical (*O*_2_^•^^−^) Scavenging Activity

**Figure 3:**
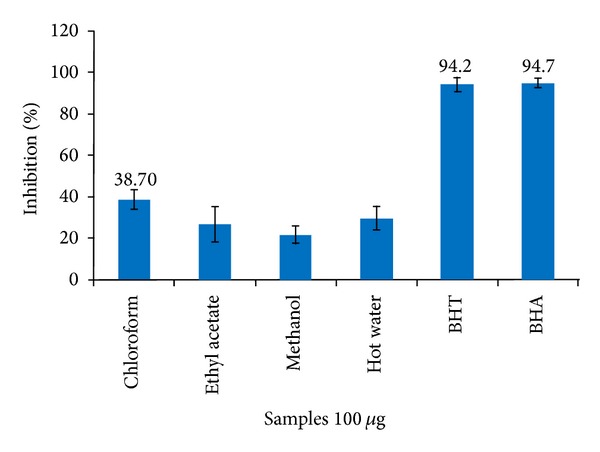
Superoxide radical scavenging activity of leaf extract of *C. dipsaceus*. Values are mean of triplicate determination (*n* = 3)  ±  standard deviation.

In Figure 3, scavenging ability of *C. dipsaceus *exhibited that the inhibition of formazan and also the percentage inhibition are directly proportional to the concentration of the plant extract. Chloroform extract of leaf was found to be higher (38.70%) while least activity of leaf (21.70%) was depicted in methanol extracts and is represented in Figure 3. Superoxide radical is known to be a very harmful species to cellular components as a precursor of more reactive oxygen species [46]. All these investigations about this harmful radical have made it possible to search for the potent superoxide scavenging natural agent which can be supplemented partially by* C. dipsaceus* as per the moderate activity.

#### 3.5.8. Phosphomolybdenum Assay

The results from Table 4 depicted that hot water extract of leaf (238.8 mg AA/g extract) has maximum activity compared to other extracts. The phosphomolybdenum assay is based on the reduction of Mo (VI) to Mo (V) by the antioxidant compounds and the formation of green phosphate/Mo (V) complex [47]. Hence, the estimation of Mo reduction activity by *C. dipsaceus* became an essential report for determining its antioxidant potential.

## 4. Conclusion

Antioxidant and nutritional properties of this plant have been evaluated for the first time. The results obtained have shown appreciable radical scavenging activity which can be taken as evidence to cure several free radical associated diseases. Nutritional contents of the leaf may also highlight the importance of this wild edible. Not only this, but the antioxidant and nutritional property of this plant together could encourage its use as a nutraceutical supplement. Further *in vivo* studies are needed to evaluate the potential of extracts and identification of the phenolic compound responsible for these properties.

## Figures and Tables

**Table 1:** Phytochemical screening of *C. dipsaceus* leaf.

Phytochemical constituents	Leaf
Carbohydrates	+++
Proteins	+++
Amino acids	+++
Alkaloids	++
Saponins	+++
Phenolic compounds	++
Tannins	++
Flavonoids	++
Glycosides	+
Flavonol glycosides	++
Cardiac glycosides	+++
Phytosterols	+++
Fixed oils and fats	+

(+): presence of chemical compound; (+) < (++) < (+++): based on the intensity of characteristic colour.

**Table:** (a) Amino acids quantification of *C. dipsaceus* leaf

Asp	Thr	Ser	Glu	Pro	Gly	Ala	Cys	Val	Met	Isoleu	Leu	Tyr	Phe	His	Lys	Arg	Gln	Trp
8.44*	3.87	3.40	10.72	3.92	4.71	5.07	1.13	5.80	1.74	4.71	7.81	3.63	5.25	1.48	4.35	4.8	ND	ND

*Values are % of protein.

**Table:** (b) Minerals, starch, and protein quantification of *C. dipsaceus* leaf

Parameters (ppm)												Starch (mg/g)	Proteins (mg/g)
N	P	K	Na	Mg	Mn	Ca	Fe	Zn	Cu	Si	B		
8900	ND	3200	ND	15300	ND	27000	24000	30.83	BDL	2000	ND	0.87	108.2

ND: not detected; BDL: below detectable level.

**Table 3:** Total phenols, flavonoids, and tannin content of leaves of *C*. *dipsaceus*.

Samples	Total phenolics (g GAE/100 g)	Tannins (g GAE/100 g)	Flavonoids (g RE/100 g)
Chloroform	5.81 ± 0.05^a^	5.49 ± 0.11^a^	4.10 ± 0.15^d^
Ethyl acetate	1.66 ± 0.62^c^	1.21 ± 0.48^c^	13.69 ± 1.44^a^
Methanol	2.62 ± 0.86^bc^	0.70 ± 0.74^d^	5.00 ± 0.52^c^
Hot water	5.52 ± 0.20^ab^	2.89 ± 0.27^b^	7.36 ± 0.19^b^

Values are mean of triplicate determination (*n* = 3) standard deviation.

GAE: gallic acid equivalents; RE: rutin equivalents.

Values followed by superscript indicate statistical significance *P* < 0.05.

**Table 4:** ABTS, FRAP, metal chelating, and phosphomolybdenum radical scavenging activity of *C. dipsaceus* leaf.

Sample extracts	ABTS (*μ*m trolox equi/g extract)	FRAP (mM Fe(II)/mg extract)	Metal chelating (g EDTA equi/100 g)	Phosphomolybdenum (mg AAE/g)
Chloroform	1811.24 ± 14.05^b^	139.21 ± 5.09^c^	1.56 ± 0.19^c^	214.3 ± 11.5^ab^
Ethyl acetate	1498.49 ± 20.25^d^	48.97 ± 1.67^d^	0.44 ± 0.03^d^	100.3 ± 8.6^d^
Methanol	1700.99 ± 20.25^c^	253.0 ± 1.86^b^	5.31 ± 0.07^b^	154.6 ± 11.9^c^
Hot water	6959.21 ± 30.93^a^	294.1 ± 1.84^a^	11.06 ± 0.44^a^	232.8 ± 1.3^a^

Values are mean of triplicate determination (*n* = 3) standard deviation.

EDTA: ethylene diamine tetraacetic acid; AAE: ascorbic acid equivalence.

Values followed by superscript indicate statistical significance *P* < 0.05.
